# New Drugs, Appliances, and Things Medical

**Published:** 1891-03-21

**Authors:** 


### NEW DRUGS, APPLIANCES, AND THINGS MEDICAL.

[All preparations, appliances, novelties, etc., of which a notice is desired, should be sent for The Editor, to care of The Manager, 140, Strand, London, W.C.]

### DR. STROCHEM'S HYPODERMIC SYRINGE.

Messrs. Polks and Co., Creechurch Lane, E.C., send us a sample of the above for notice. The syringe consists of three parts. An inner glass tube graduated in ½ CC's with the back in opaque glass so that the readings of the measures are made easy. The larger end of this is rounded and perforated with a fanhole opening; the smaller end is ground to fit the ordinary hypodermic needles. This inner tube fits air-tight into the outer one by means of a collar of vulcanized rubber tubing. The outer tube is in shape like a small thick test-tube. The advantages of this apparatus seem to us to lie in its simplicity, absence of metal fittings, the joints of which are so liable to get out of order, and the readiness with which it can be cleaned. To fill the syringe, hold the narrow end of the inner tube in the fluid to be injected and pull off, as far as is necessary, the outer tube. The fluid enters the inner tube, and having fitted a needle on and inserted it, all that remains to be done is to press the outer tube down over the inner one and the fluid is expelled. We consider that the inventor has succeeded in making a distinct advance in the mechanics of subcutaneous medication.

### KOND'S EUONYMISED COCOA.

This is a well manufactured cocoa mixed with a portion of euonymin. It is intended especially for those whose hepatic functions are in want of gentle stimulation. As is well known, euonymin is a distinct hepatic stimulant, and consequently the use of this cocoa in appropriate cases would provide a palatable and refreshing form of food and medicine at the same time.

### PETERMAN'S COCKROACH AND BEETLE FOOD (NON-POISONOUS).

Mr. J. F. Thovey, chemist, Farringdon Street, has sent us a specimen of this insect destroying food. Dr. Hassall's analysis shows its non-poisonous quality. We have submitted it to a practical test, and find that it does destroy cockroaches in large quantities, and consequently can confidently state that it is a success.

### DR. ANGELL'S FOOD FOR INFANTS AND INVALIDS.

A well-made, palatable, and nutritious food of considerable value in the feeding of those whose digestive powers are weak. It is made on the improved formula of Liebig, and contains the necessary ingredients for the building up of the tissues which are either in the process of growth or renewal after waste. We can recommend it.

